# Impact of COVID-19 on referrals to paediatric liaison psychiatry at Children's Health Ireland at Crumlin as the pandemic moved to endemic status

**DOI:** 10.1192/bjo.2024.792

**Published:** 2024-10-04

**Authors:** B. Sun, D. Adamis, F. McNicholas

**Affiliations:** Department of Child & Adolescent Psychiatry, School of Medicine and Medical Science, University College Dublin, Dublin, Ireland; Department of Psychiatry, Sligo Mental Health Services, Sligo, Ireland; Department of Paediatric Liaison Psychiatry, Children's Health Ireland at Crumlin, Dublin, Ireland; Lucena Clinic Rathgar, St John of God Hospitaller Services, Dublin, Ireland

**Keywords:** COVID-19, youth mental health, referral rates, paediatric psychiatry, public health measures

## Abstract

**Background:**

Rates of acute mental health presentations in youth were increasing pre-pandemic internationally. Longitudinal studies following COVID-19 attest to ongoing deterioration in youth mental health, recognising adverse unintended consequences following public health restrictions.

**Aims:**

To examine whether the initial post-COVID-19 increase in mental health presentations persisted following the reclassification of COVID-19 to endemic status, accompanied by the removal of most restrictions.

**Method:**

All referrals to paediatric liaison psychiatry (PLP) between January 2018 and December 2022 in a Dublin tertiary children's hospital were included in the study. An interrupted time series analysis with autoregressive integrated moving average models was conducted, examining referrals with respect to different phases of COVID-19 and application of public health restrictions.

**Results:**

Some 1385 referrals to PLP were received over the 5-year study. There was a significant decrease in PLP referrals immediately post-COVID-19, followed by a significant and sustained increase as the pandemic progressed and moved to endemic status. Public health restriction phases had a unique effect on those presenting with suicidal ideation, with a significant increase in the number of referrals. There was no effect of restrictions on other clinical profiles.

**Conclusions:**

Increased referrals for youth with mental health difficulties, reported during the COVID-19 pandemic, persisted into the early endemic stage, after COVID-19 public health restrictions ceased. Specific impacts of restrictions on suicidal ideation referrals require further study. Investment in child and adolescent mental health services remains a priority, and future pandemic response strategies need to examine unintended consequences of any enforced public health measure.

## Global burden of youth mental illness

Mental illness represents the greatest burden of disease for young people worldwide.^1^ Over the past 30 years, psychiatric disorders such as depression, anxiety, eating and conduct disorders have all increased in youth globally.^2^ Such illnesses are starting at younger ages and increasing at a faster pace when compared to adult cohorts. Longitudinal studies also report increasing rates of self-harm and suicidal ideation in children and adolescents, and by 2019, suicide was the fourth leading cause of death among 15–19 year olds worldwide^3^ and the leading cause of death among young males under 25 years in Ireland.^4^

## Impact of COVID-19 on youth mental health

During COVID-19, many countries reported further increases in rates of mental health distress and illness in youth, with rates of depression and anxiety being most noted.^5^ A Dutch longitudinal cohort study showed an increased prevalence of moderate anxiety and depressive symptoms among adolescents, with rates rising from 24.0% in 2012 and 20.2% in 2016 to 31.9% in 2020.^6^ Opportunistic longitudinal studies in England revealed increased rates of ‘probable mental health disorders’ from 10.8% in 2017 to 16.0% in 2020.^7^ Following these findings, the government and National Health Service (NHS) England implemented policies to track and support youth mental health, such as establishing school-based mental health provisions, appointing senior mental health leads and setting 4 week targets for waiting lists.^8^ A systematic review of 21 international studies generally supported the view of a deterioration in youth mental health finding.^9^ In Ireland, following an initial decline, referrals to child and adolescent mental health services (CAMHSs) showed a substantial surge, showing a 50% increase compared with pre-pandemic levels.^10^ In addition, attendances to paediatric emergency department for psychiatry reasons increased by 8.9% compared to the pre-pandemic year in Ireland.^11^

## Diverging studies and study rationale

However, not all studies have shown a deterioration in youth mental health. When adjustments are made for pre-existing trends, no increase in clinical impairment is observed in youth referred to CAMHSs, even amidst school lockdowns.^12^ Other studies have highlighted varying effects based on gender, disorder type or presence of pre-pandemic mental health vulnerabilities.^13^ Significant fluctuations have also been observed when data was examined longitudinally^14^ with speculations regarding possible impacts of various lockdown measures, especially school closures. Ireland has been recognised as having one of the most stringent lockdowns relative to other European Union countries, thus offering an opportunity to use a time series methodology to examine mental health referral rates pre- and post-COVID-19 and by public health restriction phases. A time series models the number of mental health referrals in a timeline. Using mathematical models, it is possible to predict what the number of referrals would have been like after 2020, based on pre-2020 trends. The difference between the predicted and actual number of referrals after the start of the pandemic allows us to estimate the effect of COVID-19.

The aims of this study are as follows.

1. Examine the impact of the COVID-19 pandemic on the rates of referrals to a psychiatry liaison service in a tertiary paediatric hospital in Dublin over a 5-year time period.
2. Examine whether the initial reported post-COVID-19 increase in mental health presentations persisted following the reclassification of COVID-19 to endemic status and the removal of most public health restrictions.
3. Examine the impact of the COVID-19 pandemic on certain clinical diagnoses.

We hypothesise that the upward trend of referrals seen pre-COVID-19 will continue.

## Method

This study employed a retrospective longitudinal design, utilising an electronic health record system for data collection and registry. All referrals to a Dublin-based paediatric liaison psychiatry (PLP) department between 1 January 2018 and 31 December 2022 were included in the study. Data entry was gathered by healthcare professionals, including consultants, registrars and nursing staff responsible for clinical assessments and diagnoses. All diagnoses were validated by a consultant psychiatrist to ensure accuracy. Clinical data routinely collected by the PLP included numbers of referrals, gender, reason for referral and subsequent DSM-5 diagnostic categories,^15^ and additionally the presence of suicidal ideation or self-harm behaviour.

### Diagnoses

Diagnostic categories most frequently presented were analysed: eating disorders, anxiety disorders, major depressive disorder (MDD), oppositional disorder, attention deficit hyperactivity disorder (ADHD) and autism spectrum disorder (ASD). A diagnosis of ‘low mood’ was also included in our analysis, as many youth presented with transient feelings of sadness and anhedonia, but insufficient to meet the DSM 5 criteria for MDD. The presence of suicidal ideation and self-harm was also included as it represented a significant number of presentations across many different diagnoses and with some studies suggesting increases post-pandemic. ‘Suicidal ideation’ refers to any self-reported thoughts about ending one's life (active) or one's life being over (passive). ‘Self-harm’ is defined as any act of self-injury, irrespective of intent. This can include actions both with and without suicidal intent. The combined suicidal ideation/self-harm group includes any individuals who exhibit either suicidal ideation or engage in self-harm behaviours, or both. Identifying individuals within this group is critical, as it often indicates a higher risk and complexity of psychiatric profile, warranting more intensive clinical interventions.

### COVID-19 and restrictions

Data regarding the onset of COVID-19 and the subsequent restrictions in the Republic of Ireland (ROI) were sourced from the ROI Citizens COVID-19 information website,^16^ accessed on 14 March 2023. This information outlined the following phases.

1. Phase 0: Pre-COVID-19 (1 January 2020 to 29 February 2020).
2. Phase 1: Initial restrictions including the lockdown (1 March–31 May 2020), marking the closure of schools, colleges and non-essential businesses.
3. Phase 2: Reinstitution of restrictions and school closures, spanning 1 January–28 February 2021.
4. Phase 3: Further periods of restriction primarily affecting holiday social gatherings and postponed school re-opening: 3 December 2021–30 January 2022.

The COVID-19 period was defined accordingly from 1 March 2020 to 28 February 2022.^16^

Ethics: This study was conducted in accordance with the Declaration of Helsinki. An ethics waiver was received by the hospital ethics' committee, given only anonymised data used.

### Statistical analysis

An autoregressive integrated moving average (ARIMA) model, ideal for time series data showcasing trend, autocorrelation, heteroscedasticity and stationarity, was employed.

This study encompassed 5 years of measurements with two events (interruptions): the emergence of COVID-19 and the implementation of related restrictions (discrete event). The pandemic's onset was abrupt, with enduring effects (from 1 March 2020 to 28 February 2022), following a step function. The restrictions, while also abrupt, had more transient impacts, hypothesised to align with an extended pulse function given their repeated three-time occurrence.

ARIMA models were conducted to examine the effects of the events on certain dependent variables such as number of referrals, gender-based referrals and reason-based referrals. IBM SPSS for Windows, version 25.0 software was utilised. An algorithm was applied to determine and estimate the optimal ARIMA model via the SPSS modeler command. Finally, the Ljung–Box test was used to assess the residuals for normality.

## Results

1. Descriptive statistics

Data were collected over a 5-year period from 1 January 2018 to 31 December 2022. Table 1 shows the number of referrals, demographics, number of people referred with a diagnosis of eating disorders, anxiety disorders, MDD, low mood, oppositional disorder, ADHD, ASD and the presence of suicidal ideation and self-harm. The total number of referrals pre-COVID-19 (1 January 2018–29 February 2020) was 488, resulting in a mean monthly referral of 18.8; the total number post-COVID-19 (1 March 2020–31 December 2022) was 897, corresponding to a mean monthly referral of 26.4. The age range is from 1 to 20 years old.

**Table 1:** Descriptive statistics of the sample

Year	Referrals			Age		Male	Female	Suicidal ideation	Self-harm	Suicidal ideation/self-harm	Eating disorders	Anxiety disorders
	*n* (%)	Mean	s.d.	Mean	s.d.	*n* (%)	*n* (%)	*n* (%)	*n* (%)	*n* (%)	*n* (%)	*n* (%)
2018	233 (16.8)	19.42	4.81	13.31	0.82	92 (21.6)	141 (14.7)	54 (9.5)	55 (8.5)	90 (10.8)	18 (9.2)	41 (18.3)
2019	219 (15.8)	18.25	5.43	13.58	0.57	83 (19.5)	136 (14.2)	97 (17.1)	77 (11.9)	119 (14.3)	22 (11.3)	38 (17.0)
2020	294 (21.2)	24.50	6.26	14.18	0.35	87 (20.4)	207 (21.6)	98 (17.3)	175 (27.0)	192 (23.0)	50 (25.6)	39 (17.4)
2021	363 (26.2)	30.25	6.77	13.74	0.44	92 (21.6)	271 (28.3)	171 (30.1)	194 (29.9)	245 (29.4)	55 (28.2)	48 (21.4)
2022	276 (19.9)	23.00	8.72	13.79	0.50	72 (16.9)	204 (21.3)	148 (26.1)	148 (22.8)	187 (22.4)	50 (25.6)	58 (25.9)
**Total**	1385 (100)	23.08	7.63	13.72	0.61	426 (100)	959 (100)	568 (100)	649 (100)	833 (100)	195 (100)	224 (100)

**Fig. 1:**
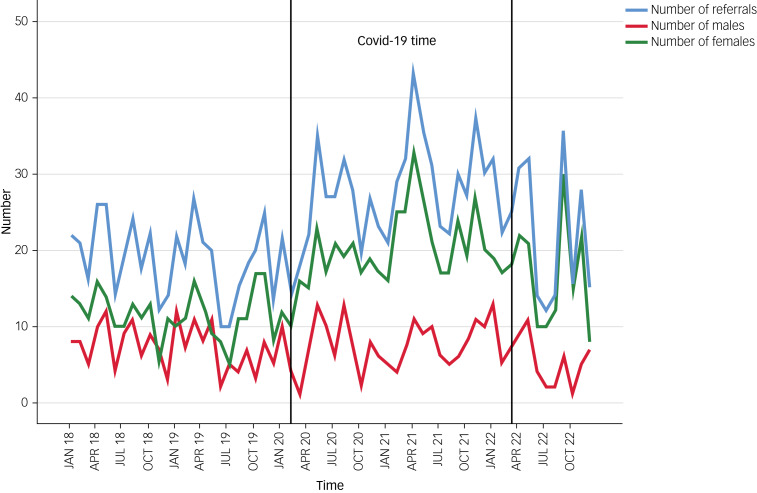
Number of referrals, number of males and females in monthly intervals from January 2018 to December 2022.

**Fig. 2:**
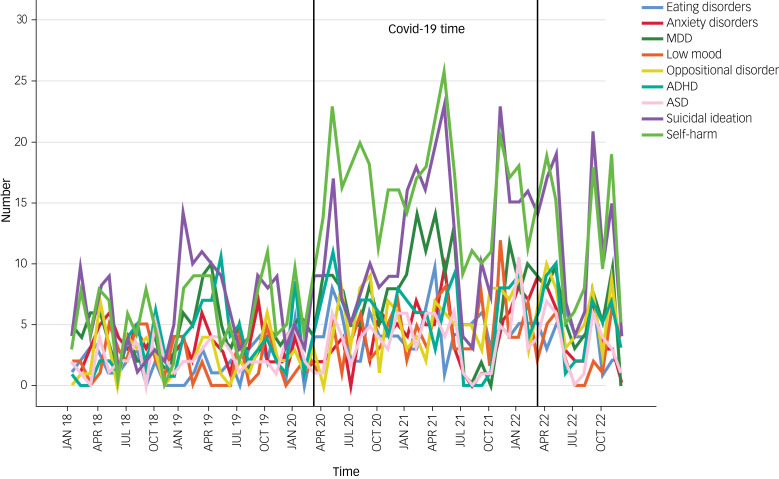
Number of referrals per diagnostic category. MDD, major depressive disorder; ADHD, attention deficit hyperactivity disorder; ASD, autism spectrum disorder.

Figure 1 shows the number of referrals and the number of referrals of each gender at each month from January 2018 to December 2022. The two vertical lines indicate the beginning and end of the COVID-19 pandemic in the ROI, and Fig. 2 demonstrates the number of referrals per diagnostic category.

1. Time series (Note: parameter estimates of each model are shown in Table 2. Graphs and additional statistics related to model fit are shown in Supplementary Information Document available at https://doi.org/10.1192/bjo.2024.792)

**Table 2:** Synoptic table of the estimates (estimate: magnitude of impact-slope) with significant levels of each of the above models

Model	Variables	Estimate	s.e.	*t*	Significance	95% CI
Referrals ARIMA (0,0,0) (1,1,0)	Constant	1.002	0.683	1.467	0.149	0.834–1.35
	COVID-19 pandemic	8.857	1.526	5.805	**<0.0001**	6.04–11.67
	Restrictions	−5.157	2.614	−1.973	0.055	−7.68–3.70
Female gender ARIMA (3,1,1) (0,0,0)	Constant	0.111	0.030	3.632	0.001	0.058–0.162
	COVID-19 pandemic	6.385	1.279	4.991	**<0**.**0001**	4.43–8.51
	Restrictions	−0.849	1.948	−0.436	0.665	−4.35–2.66
Male gender ARIMA (0,0,0) (3,1,0)	Constant	−0.375	0.308	−1.215	0.231	−0.78–0.02
	COVID-19 pandemic	1.800	0.696	2.588	**0.013**	0.54–3.05
	Restrictions	−2.216	1.338	−1.656	0.105	−4.769–0.346
Eating disorder ARIMA (0,1,1) (0,0,0)	Constant	0.048	0.017	2.868	0.006	0.018–0.075
	COVID-19 pandemic	1.581	0.595	2.656	**0.010**	0.416–2.66
	Restrictions	−0.166	0.864	−0.192	0.848	−1.63–1.57
Anxiety disorders ARIMA (0,0,2) (0,0,0)	Constant	3.574	0.598	5.975	<0.0001	2.447–4.702
	COVID-19 pandemic	−0.125	0.946	−0.132	0.895	−1.978–1.723
	Restrictions	1.313	0.968	1.356	0.181	−0.635–3.269
Major depressive disorder (MDD) ARIMA (1,0,0) (0,0,0)	Constant	4.913	0.781	6.291	<0.0001	3.434–6.391
	COVID-19 pandemic	1.666	1.300	1.282	0.205	−0.802–4.133
	Restrictions	2.096	1.570	1.335	0.187	−0.909–5.096
Low mood ARIMA (0,0,0) (0,0,0)	Constant	2.222	0.377	5.891	<0.0001	1.501–2.942
	COVID-19 pandemic	2.719	0.666	4.082	**<0**.**0001**	1.446–3.99
	Restrictions	−1.798	1.016	−1.769	0.082	−3.740–0.143
Oppositional disorder ARIMA (1,0,0) (0,0,0)	Constant	3.317	0.508	6.535	<0.0001	2.355–4.279
	COVID-19 pandemic	2.082	0.881	2.363	**0.022**	0.387–3.775
	Restrictions	−0.951	1.252	−0.759	0.451	−3.377–1.477
ADHD ARIMA (1,0,0) (0,0,0)	Constant	3.949	0.631	6.253	<0.0001	2.752–5.144
	COVID-19 pandemic	0.856	1.072	0.798	0.428	−1.173–2.886
	Restrictions	2.446	1.395	1.754	0.085	−0.219–5.111
Autism spectrum disorder ARIMA (0,0,1) (0,0,0)	Constant	2.635	0.390	6.757	<0.0001	1.892–3.413
	COVID-19 pandemic	0.954	0.669	1.426	0.160	−0.422–2.221
	Restrictions	−0.209	0.963	−0.217	0.829	−0.107–2.867
Suicidal ideation ARIMA (1,0,0) (1,0,0)	Constant	7.307	1.839	3.973	<0.0001	4.269–11.209
	COVID-19 pandemic	2.595	2.105	1.233	0.223	−0.895–7.148
	Restrictions	3.736	1.821	2.052	**0.045**	−1.617–5.954
Self-harm ARIMA (1,0,0) (0,0,0)	Constant	7.555	1.090	6.933	<0.0001	5.471–9.639
	COVID-19 pandemic	7.831	1.842	4.251	**<0**.**0001**	4.197–11.458
	Restrictions	0.644	2.303	0.280	0.781	−3.716–5.006

Bold indicates statistical significance. ARIMA, autoregressive integrated moving average; ADHD, attention deficit hyperactivity disorder.

**Fig. 3:**
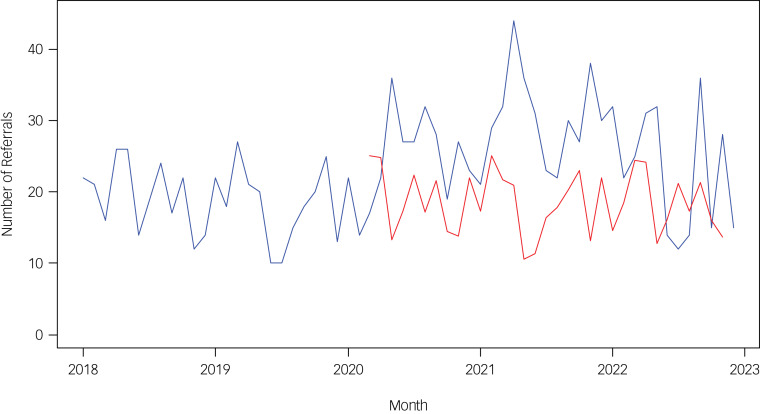
Forecasting versus actual referrals. Note: blue line represents the actual number of referrals per month, while red line represents the forecasting number of referrals based on the previous years.

Figure 3 illustrates the impact of COVID-19 on monthly referrals. The red line represents the forecasted number of referrals based on data from previous years, assuming COVID-19 had not occurred. The blue line shows the actual number of referrals per month during and after the COVID-19 pandemic. It is evident that referrals increased during COVID-19 and remained elevated in the post-COVID era, although to a lesser extent.

- *Number of referrals*: Different ARIMA models were used to examine any difference on number of referrals consequent to COVID-19. The best fitting model was ARIMA (0,0,0) (1,1,0) with *R*^2^ = 30.2% and BIC (Bayesian information criterion) = 4.177 (lower BIC means better model among other alternative modes) (Table 2). This shows a significant increase in referrals during the COVID-19 pandemic (parameter estimate = 8.857). However, the phases of COVID-19 restrictions did not have any significant effect in the number of referrals to the PLP service. Figure 3 illustrates the effect of COVID-19 on monthly referrals.
- *Female genders*: The best fitting model was ARIMA (3,1,1) (0,0,0) with *R*^2^ = 56.5% and BIC = 3.38. This model shows that COVID-19 had a significant effect on the number of females presenting to PLP. The phases of COVID-19 restrictions did not have a significant effect.
- *Male genders*: The best fitting model was ARIMA (0,0,0) (3,1,0) with *R*^2^ = 5.7% and BIC = 2.930. This model shows that COVID-19 had a significant effect on the number of males presenting to PLP. The phases of COVID-19 restrictions did not have a significant effect.
- *Number of presentations with a diagnosis of eating disorder*: The best fitting model was ARIMA (0,1,1) (0,0,0) with *R*^2^ = 40.6% and BIC = 1.506. This model shows that COVID-19 had a statistically significant effect on the number of eating disorder diagnoses. The phases of COVID-19 restrictions did not have a significant effect.
- *Number of presentations with a diagnosis of anxiety disorders*: The best fitting model was ARIMA (0,0,2) (0,0,0) with *R*^2^ = 22.9% and BIC = 1.84. Neither the COVID-19 pandemic nor associated phases of restrictions had any statistically significant effect on the number of PLP presentations with anxiety disorders.
- *Number of presentations with a diagnosis of MDD*: The best fitting model was ARIMA (1,0,0) (0,0,0) with *R*^2^ = 26.5% and BIC = 2.572. Neither the COVID-19 pandemic nor associated phases of restrictions had any statistically significant effect on the number of PLP presentations with MDD.
- *Number of presentations with a diagnosis of low mood*: The best fitting model was ARIMA (0,0,0) (0,0,0) with *R*^2^ = 22.6% and BIC = 1.838. This model shows that COVID-19 had a statistically significant effect on the number of presentations with low mood. The phases of COVID-19 restrictions did not have significant effect.
- *Number of presentations with a diagnosis of oppositional disorder*: The best fitting model was ARIMA (1,0,0) (0,0,0) with model *R*^2^ = 14.5% and BIC = 2.202. This model shows that COVID-19 had a statistically significant effect on the number of presentations of people with oppositional disorder diagnoses. The phases of COVID-19 restrictions did not have a significant effect.
- *Number of presentations with a diagnosis of ADHD*: The best fitting model was ARIMA (1,0,0) (0,0,0) with *R*^2^ = 19.8% and BIC = 2.363. Neither the COVID-19 pandemic nor associated phases of restrictions had any statistically significant effect on the number of PLP presentations with ADHD.
- *Number of presentations with a diagnosis of ASD*: The best fitting model was ARIMA (0,0,1) (0,0,0) with *R*^2^ = 32.6% and BIC = 1.556. Neither the COVID-19 pandemic nor associated phases of restrictions had any statistically significant effect on the number of PLP presentations with ASD.
- *Number of presentations with suicidal ideation*: The best fitting model was ARIMA (1,0,0) (1,0,0) with *R*^2^ = 58.1% and BIC = 3.228. This model shows that COVID-19 did not have a statistically significant effect on the number of presentations of people with suicidal ideation. However, the phases of COVID-19 restrictions had a significant effect on the number of suicidal ideation presentations.
- *Number of presentations with self-harm*: The best fitting model was ARIMA (1,0,0) (0,0,0) with *R*^2^ = 50.1% and BIC = 3.32. This model shows that COVID-19 had a statistically significant effect on the number of presentations of people with self-harm presentations. The phases of COVID-19 restrictions did not have significant effect.

## Discussion

The data presented in Table 1, alongside Figs. 1 and 2, provide a comprehensive overview of referral patterns to our PLP service from 1 January 2018 to 31 December 2022. Table 1 highlights a substantial increase in the total number of referrals over the 5-year period with an escalation post the emergence of COVID-19, underscoring the profound impact of the pandemic on children's mental health. This increase is mapped on a month-by-month basis in Fig. 1, which shows three sizable spikes around May 2020, April 2021 and December 2021. These align with three periods of intensified public health measures and societal disruptions, including lockdowns and transitions in educational settings in Ireland. Furthermore, Fig. 2 expands on this analysis by categorising referrals by diagnostic criteria, revealing notable increases in self-harm, suicidal ideation and eating disorders during the pandemic. The authors speculate that this rise may be indicative of the heightened stress and isolation experienced by children during lockdown periods.

Using time series analysis, we observed that the COVID-19 pandemic had a significant effect on the overall number, gender distribution and clinical presentations of referrals. Overall numbers were increased as was the number of females and males referred to the PLP post pandemic onset. There was also an effect found with an increase in the number presenting with eating disorders, low mood, self-harm and oppositional disorder. This aligns with evidence from other Irish studies, showing increased mental health attendances^11^ and admissions^17^ despite reductions in general paediatric attendances for all other reasons.

Hernandez-Calle et al^18^ conducted a very similar study, also using interrupted time series analysis, examining rates of referrals to a Madrid paediatric emergency department pre- and post-COVID-19. They reported stable pre-COVID-19 referrals (October 2018–February 2020), followed by a significantly sustained increase, post-COVID-19, up to April 2021. They reported a specific increase in referrals for suicide-related phenomena, anxiety and depression. Unlike many other studies, they did not find any increase in referrals for eating disorders. Our study supports and extends these findings, with a larger cohort and a longer duration of study. Consistent with Hernandez-Calle et al, we also found an increase in post-COVID-19 referrals, which continued until December 2022, but did not find any effect on referrals for anxiety or depression. Consistent with many national and international studies,^17,19,20^ and at odds with the Spanish cohort, we found an increase in referrals of eating disorders. To what extent these differences reflect difference in access to other services during COVID-19 has not been examined. These increases were over and above what would have been expected by a continuation of the pre-COVID-19 upward trend (Fig. 3).

In addition, a number of systematic reviews examining international data indicated increased rates of general depression symptoms^21^ and self-harm during COVID-19. However, published data suggest a stronger increase for females than males,^22^ in contrast to our finding, which indicates an increase for both genders. However, this increase in both genders may be because of the overall increased number of referrals experienced during COVID-19. A UK study reported self-rated psychological problems, depression and functional impairment, and psychiatric referrals appeared higher among youths after school re-opening post lockdowns.^12^ The ROI and New Zealand have reported increases in eating disorders, with descriptions of a ‘tsunami-like' increase of presentations with eating disorders,^17,19,23^ which are consistent with our results. Also, we find the COVID-19 pandemic did not result in any significant effect on the number of referrals with suicidal ideation alone, or the number of youth presenting with other diagnostic disorders, such as anxiety, ADHD, ASD and major depression. Similar findings of reduced referrals for behavioural difficulties and ADHD have been reported when examining general practitioner (GP) referrals to, and attendance at, CAMHSs.^24^ Rates of ASD admissions to paediatric hospitals have also been reported to be reduced.^17^ This finding can be attributed to the decreased visibility of symptoms to external observers, as social restrictions could have led to fewer opportunities for teachers and coaches at school who typically observe and refer youth for these issues to interact with them, potentially underreporting or delaying recognition of symptoms. In addition, the limited social interactions during lockdowns might have made it less likely for exacerbations in symptoms that typically prompt hospital admissions to occur.

The finding of a lack of specific impact of the COVID-19 phases of restrictions on self-harm or psychiatric disorder type, such as depression or eating disorders, does not imply that restrictions did not have an effect on the genesis of illness and help seeking, but rather it was not observable at the time the restrictions were in place. It also recognised that disaster-induced psychological impacts are often delayed. Whilst onset of various disorders may commence during restrictions and be triggered by them, presentation to services may be delayed. This may be linked to ‘stay at home’ requests or the gradual emergence of symptoms to reach levels of impairments sufficient for help seeking. For example, presentation of eating disorders may be delayed until significant weight has been lost, leading to medical compromise. Equally, the emergence of low mood, reduced social interest or poor attention may be perceived by parents as understandable, expected or justified during periods of restrictions, assumed to recover post restrictions, and hence help seeking delayed until restrictions are removed but obvious impairment persists. Anxiety disorders triggered by restrictions may only reach a threshold for presentation following return to school and/or social re-engagement. This might explain why over the period of COVID-19, presentations of eating, depressive and anxiety disorders were elevated, but not specifically linked to restriction phases. There is already published evidence that for youth with premorbid social anxiety, school closures were linked with improvement in youth mental health.^25^

There is a variety of reasons for the increased number of referral and clinical presentations owing to COVID-19 and the associated restrictions. Childhood and adolescence represent sensitive developmental periods, and prolonged deprivations of personal, educational and societal liberties are now recognised to have had significant adverse effects.^26,27^ This in part was reflected by findings from studies showing higher rates of referrals as restrictions were lifted and access re-opened,^27^ and higher referral rates following school re-opening and re-establishing academic and peer contact after a period of absence.^12^

The significant psychological impact of COVID-19 and associated restrictions have been well documented.^14^ Many countries examined the lived experiences of youth during COVID-19 and the impact of COVID-19 restrictions. Large cohorts (>20 000) of youth reported decreasing levels of mental health well-being as the pandemic progressed, particularly in anxiety and depression. Youth reported increased worry, anxiety, depression and concerns related to missing friends and school.^28,29^ Others reported increased difficulties linked to school return and difficulty accessing specialist services.^28,30^ Qualitative studies highlight feelings of social isolation, depression and anxiety, and increases in maladaptive behaviour during restrictions, and families of children with ASDs reported increased mental health difficulties linked to changes in routine.^31^

Beyond the direct effects of the pandemic, increased family interactions during prolonged periods at home may have also contributed to the rise in mental health referrals. As families spent more time together owing to public health restrictions, parents and caregivers became more aware of changes in their children's behaviours and emotional states, potentially leading to greater recognition and reporting of mental health concerns. Parenting stressors relating to working from home, addressing educational needs of children and lack of suitable working space, may all have led to heightened expressed emotions. In addition, the intensified family dynamics and reduced access to external support systems during lockdowns could have exacerbated existing issues, further driving the demand for paediatric mental health services. Moreover, evidence from this period reveals that parental anxiety and depression became increasingly prevalent during lockdowns,^32^ with the findings of a direct correlation between financial strains and parental mental health, which indirectly, yet significantly, influenced the mental well-being of their children.^32^

Notwithstanding adverse mental health impacts for many, there have been a number of children whose mental health improved during the pandemic. Surprisingly, both UK and US studies have reported better outcomes in those with pre-existing mental health difficulties.^33,34^ It is possible that for some working from home facilitated lower parental stress and increased quality time with parents; school closures and online learning may have reduced certain school-related stressors and social anxiety. What is less certain is whether any initial benefits persisted as the restrictions waxed and waned, schools re-opened and closed, financial aspects of reduced or altered working hours changed and whether resilience tanks ran dry. Even in studies reporting an average reduction in mental health outcomes in their cohorts, there are subgroups who did well, ranging from 8% to 30%, depending on whether self- or parent-rated.^33^

Examining the impact of public health measures in addition to the impact of the pandemic is important to ensure that future public health measures, when enforced, are done so in an informed manner, weighing the balances for and against associated risks. For example, Ludwig-Walz et al^21^ systematically examined levels of depression among children and adolescents across Europe. They found a post-pandemic increase in depression symptoms in general but noted that this increase was more pronounced when public health measures were more stringent or associated with school closures. This suggests that social distancing policies enforced as part of the pandemic containment may have had specific negative impacts on youth. They also reported that public health measures had a higher impact among males than females, possibly linked to lower levels of depression in males pre-pandemic. This might have relevance to our study, which found a pandemic-related increase in overall numbers specific to both female and male gender, but we did not find any gender-specific effect when examining COVID-19 restrictions. Neither the pandemic onset nor restrictions had any specific impact on MDD. Taking pre-pandemic trends and trajectories into account in study analysis is also crucial.

### Limitations

Strengths of the study include the extended period of data collection over a 5-year period and comprehensive data capture of all referrals to a psychiatry liaison service in a major paediatric hospital, assessed by clinicians. However, there are notable limitations. These include a lack of granular data regarding the varied impacts of public health restrictions and the pandemic on youth, especially in relation to their pre-existing mental health status. In addition, the study could not link patients with prior presentations at other locations. Also, owing to the specific nature of hospital emergency department attendance, the findings might not be generalisable to the broader population.

### Clinical significance

Before COVID-19, referrals to CAMHSs in Ireland, as in other countries, were increasing.^35^ Service planning and improvements have been slow across Europe, with concerns that the CAMHS has not been prioritised^36^ and this cohort of youth has been referred to as ‘the neglected quarter’.^37^ The increased demand attributable to COVID-19 and associated restrictions on already over-stretched services will create a new crisis. Although some countries, such as the UK, have pledged to prioritise youth mental health and create a suite of accessible services for youth, the current reality of long waiting lists and over-reliance on emergency departments for mental health crises remain. COVID-19-specific increases in referrals will place additional demand on services, exposing staff already stressed to even higher levels of burnout and occupational stress.^38^ These factors have been directly implicated in difficulties with staff retention and recruitment.^39^

The nuanced nature of COVID-19 and the impact on the mental health of young people have highlighted a complex picture, exposing already known inequalities in social determinants of health, with those most vulnerable having been most affected. This has led some authors to describe COVID-19 as a syndemic,^40^ recognising that the pandemic impact has not been indiscriminate but has interacted with other risk factors, including service-related deficits in CAMHSs.

Despite the declaration by the World Health Organization (WHO) On 5 May 2023, and more than 3 years later, to downgrade the pandemic from a public health emergency of international concern,^41^ ongoing surveillance of youth mental health is warranted, as is prioritisation of service development in CAMHSs. Both should be seen as a public health imperative.

## Supporting information

Sun et al. supplementary material Sun et al. supplementary material

## Data Availability

Data supporting this study are available from the corresponding author.
