# Supplementary material for: Impact of COVID-19 on referrals to paediatric liaison psychiatry at Children's Health Ireland at Crumlin as the pandemic moved to endemic status
Source: BJPsych Open. 2024 Oct 4;10(5):e171. doi: 10.1192/bjo.2024.792 (PMC11536224; doi:10.1192/bjo.2024.792)
Supplement: Sun et al. supplementary material [file S2056472424007920sup001.docx]

**Supplemental Information**

Graphs for the models and Ljung-Box tests (Residual ACF, Residual PACF, Observed vs Fitted, Examination of Residual’s Distribution)

a) Number of referrals


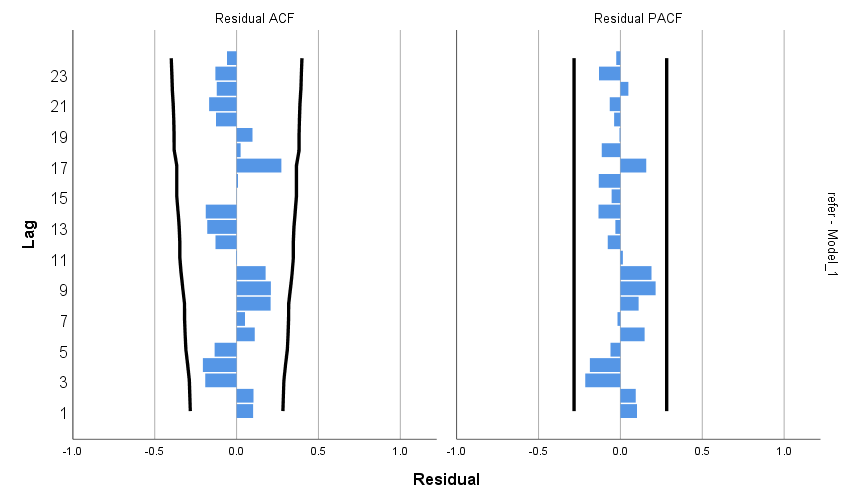

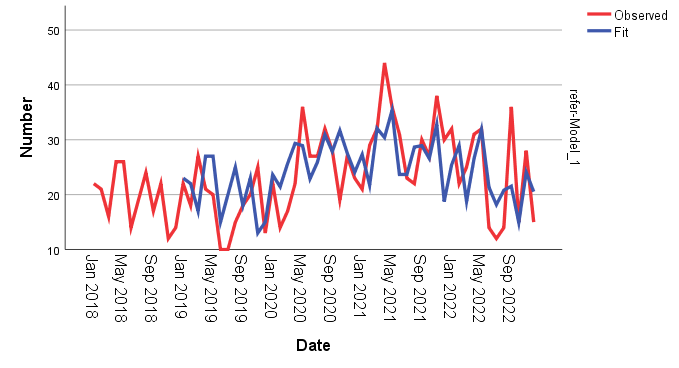


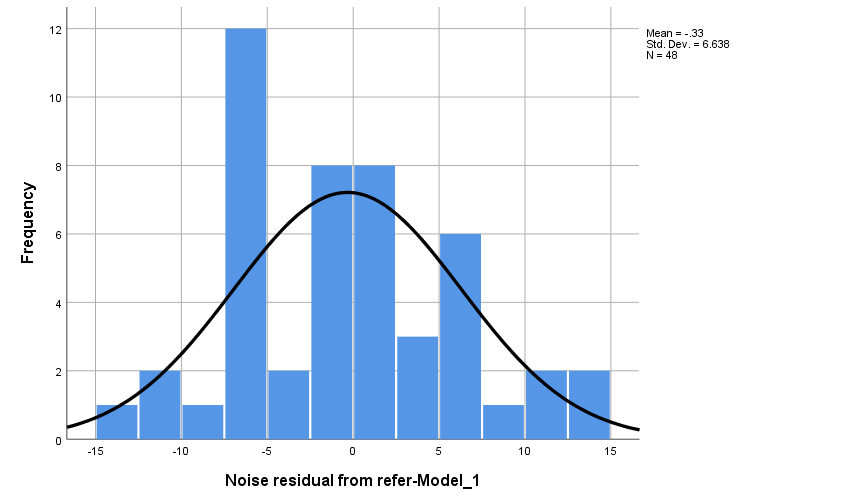

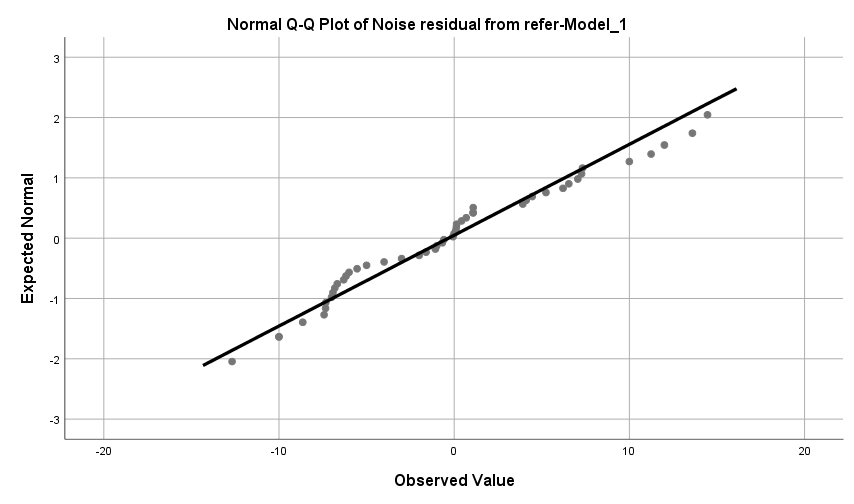


| Model | Number of Predictors | Model Fit statistics | Ljung-Box Q | | | Number of Outliers |
| --- | --- | --- | --- | --- | --- | --- |
|  |  | Stationary R-squared | Statistics | DF | Sig. |  |
| Number of referrals | 2 | .552 | 26.127 | 17 | .072 | 0 |

b) Female

**
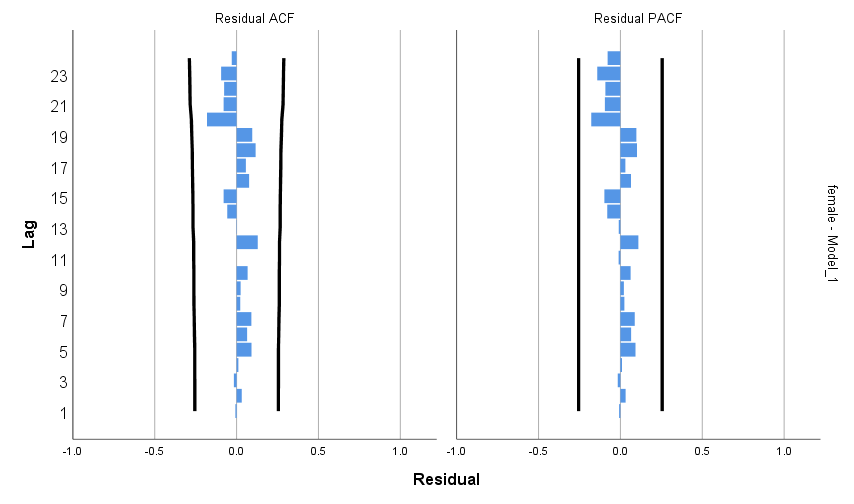

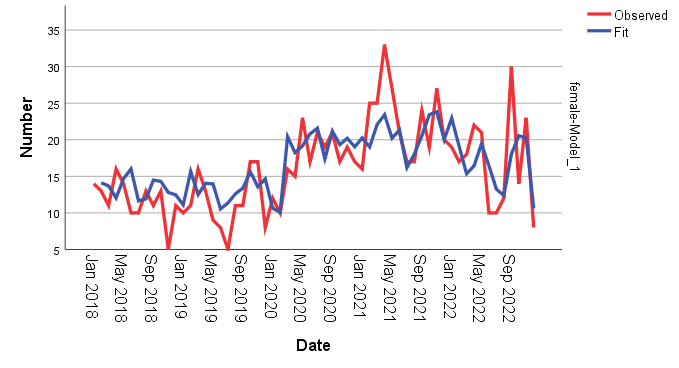
**

**
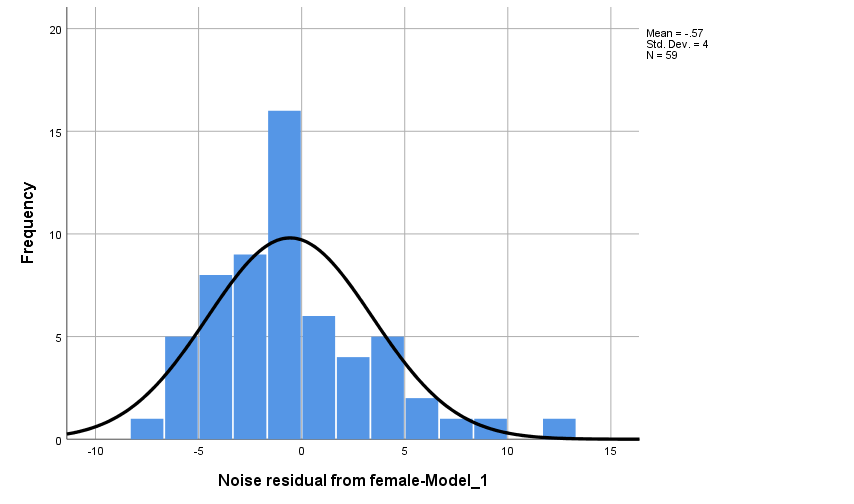

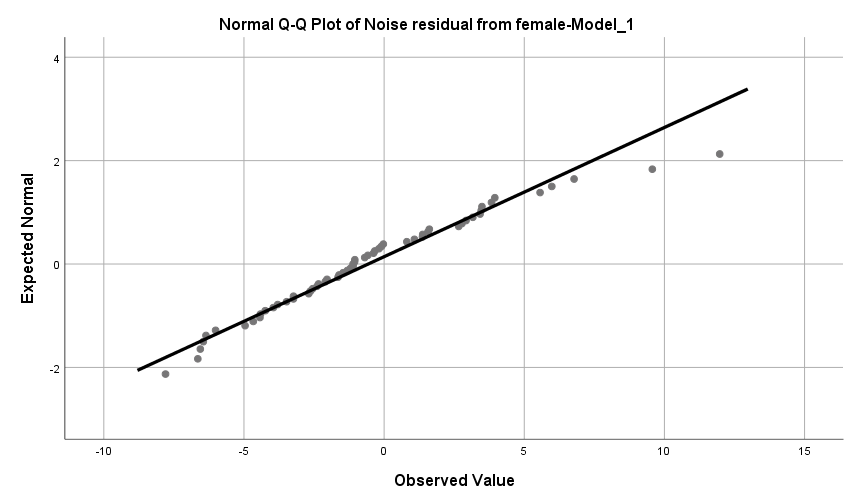
**

| Model | Number of Predictors | Model Fit statistics | Ljung-Box Q(18) | | | Number of Outliers |
| --- | --- | --- | --- | --- | --- | --- |
|  |  | Stationary R-squared | Statistics | DF | Sig. |  |
| female | 2 | .536 | 5.911 | 14 | .969 | 0 |

c) Male

**
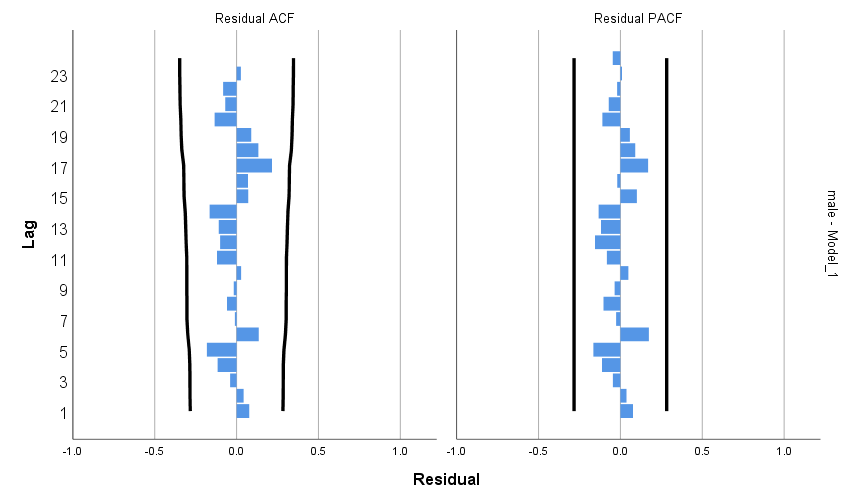

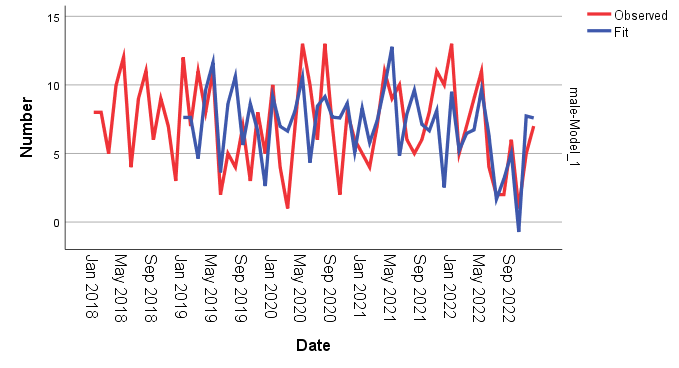
**

**
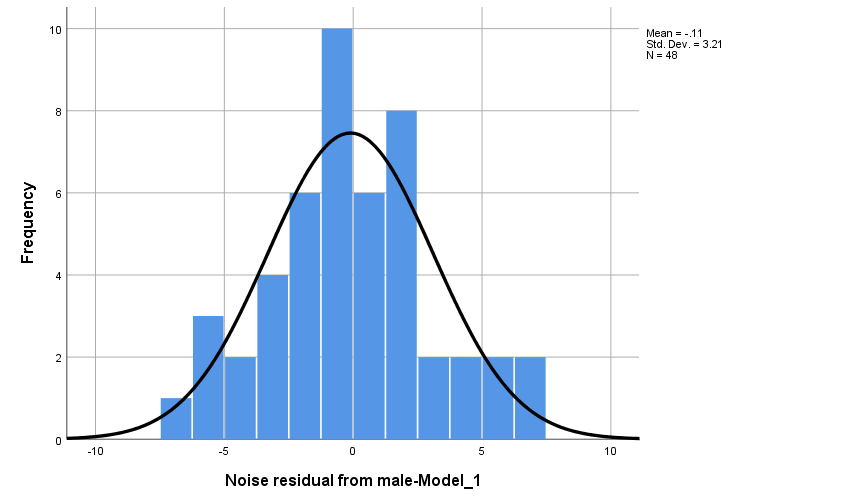

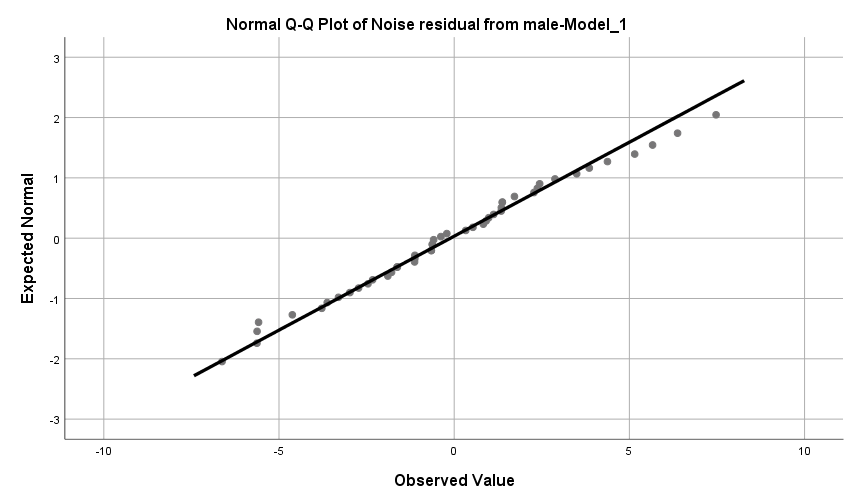
**

| Model | Number of Predictors | Model Fit statistics | Ljung-Box Q(18) | | | Number of Outliers |
| --- | --- | --- | --- | --- | --- | --- |
|  |  | Stationary R-squared | Statistics | DF | Sig. |  |
| Male | 2 | .445 | 14.502 | 15 | .488 | 0 |

d) Eating disorder

**
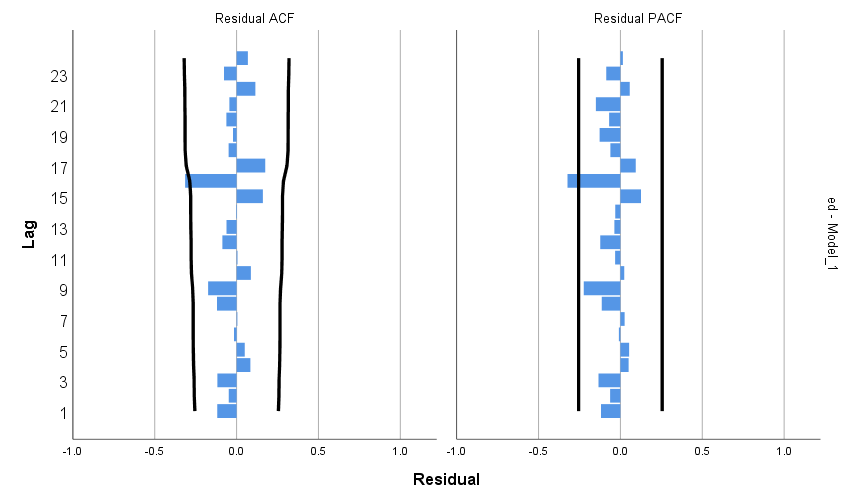

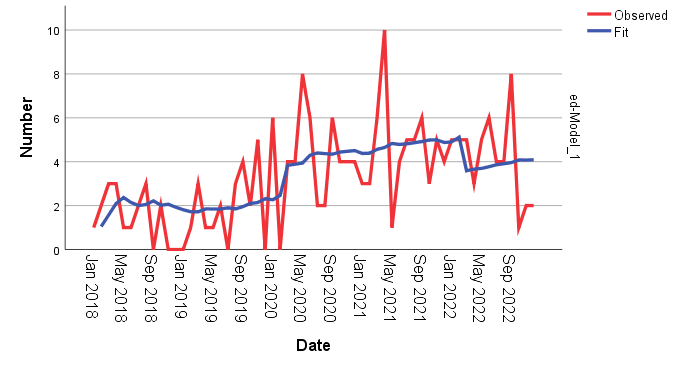
**

**
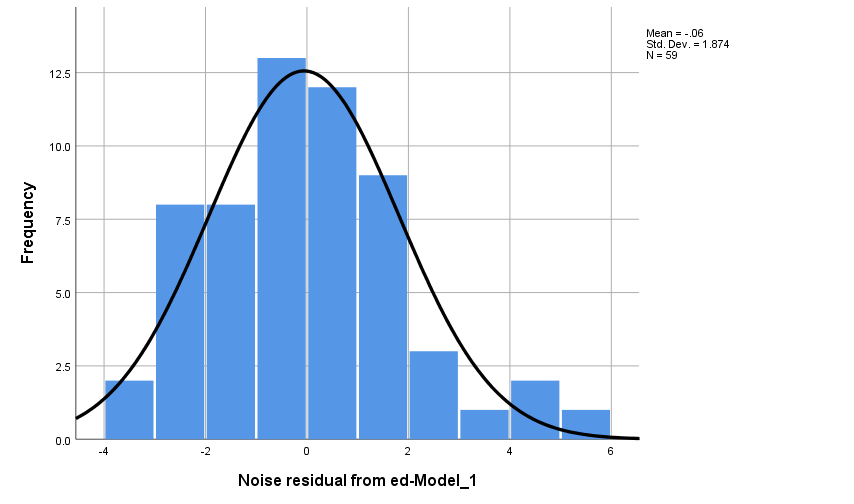

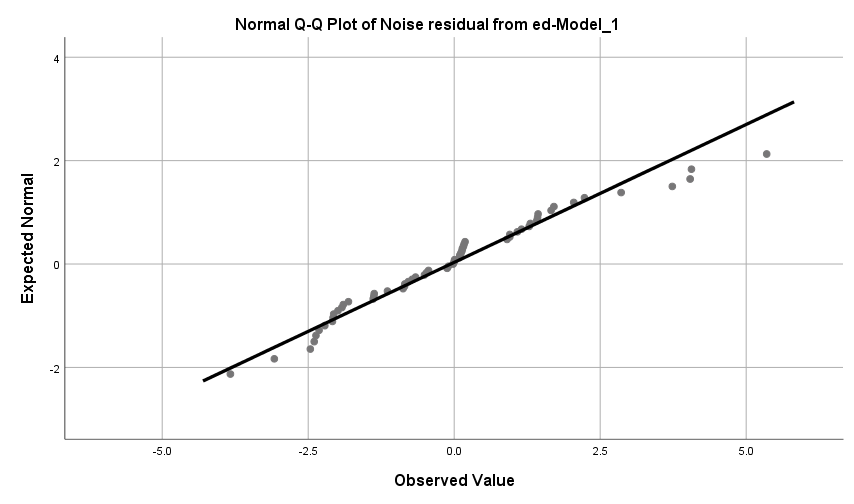
**

| Model | Number of Predictors | Model Fit statistics | Ljung-Box Q(18) | | | Number of Outliers |
| --- | --- | --- | --- | --- | --- | --- |
|  |  | Stationary R-squared | Statistics | DF | Sig. |  |
| Eating Disorders | 2 | .540 | 20.407 | 17 | .254 | 0 |

e) Anxiety disorders


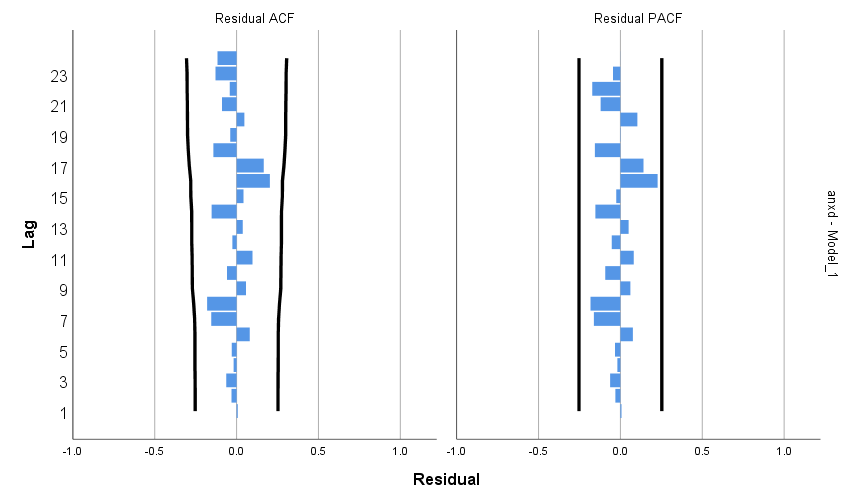

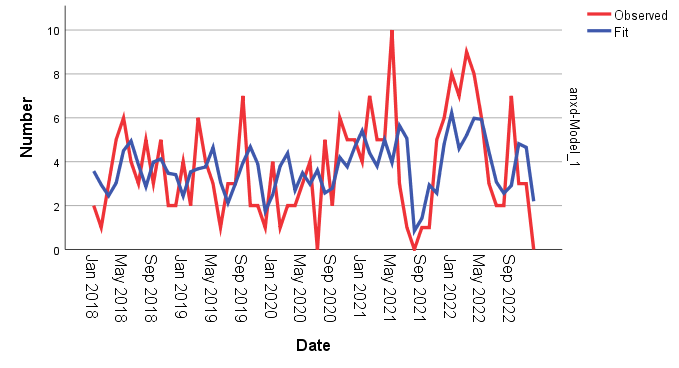


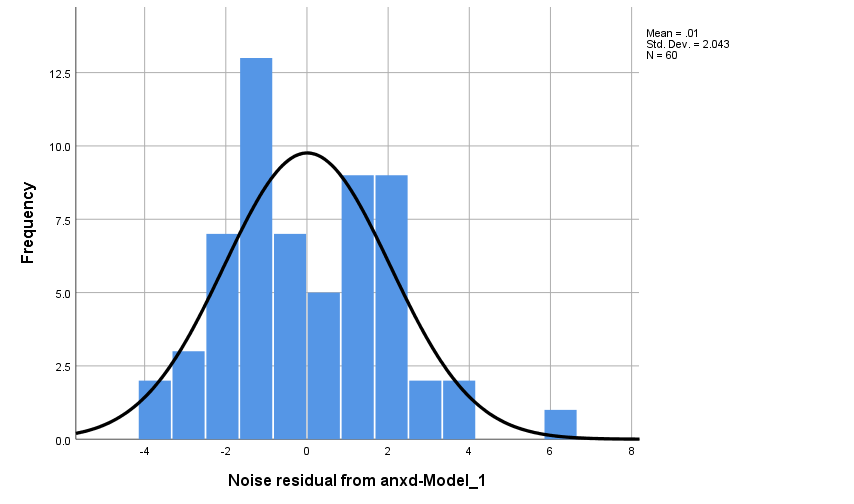

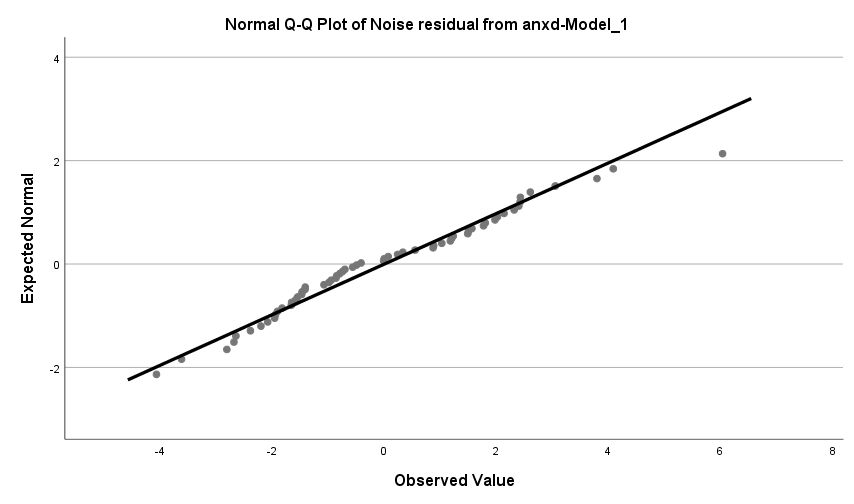


| Model | Number of Predictors | Model Fit statistics | Ljung-Box Q(18) | | | Number of Outliers |
| --- | --- | --- | --- | --- | --- | --- |
|  |  | Stationary R-squared | Statistics | DF | Sig. |  |
| Anxiety Disorders | 2 | .229 | 15.922 | 16 | .458 | 0 |

f) Major Depressive Disorder (MDD)


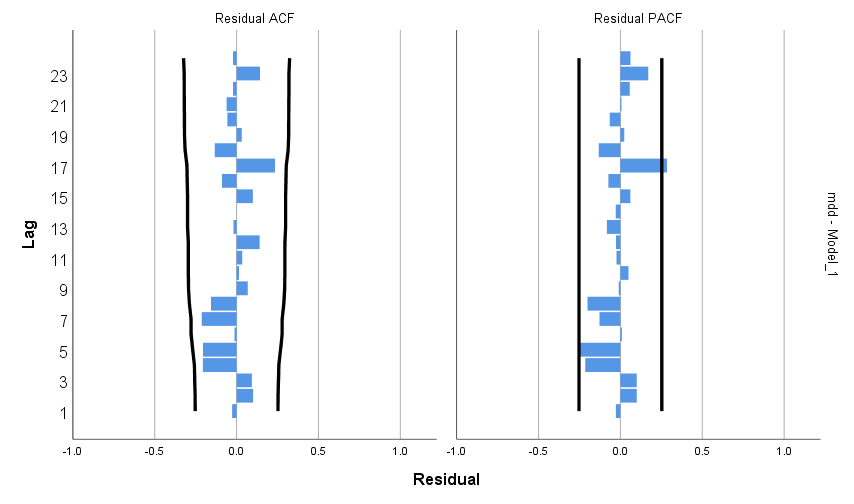

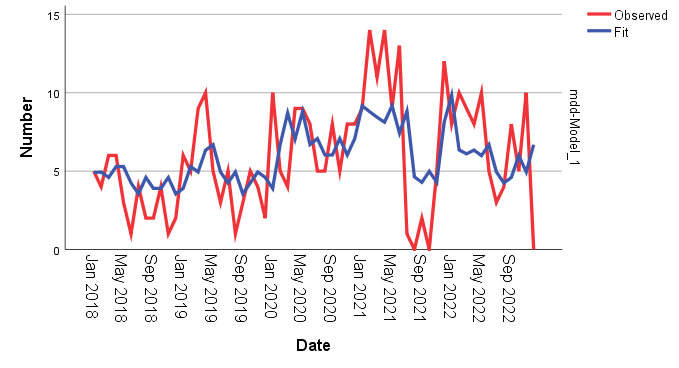


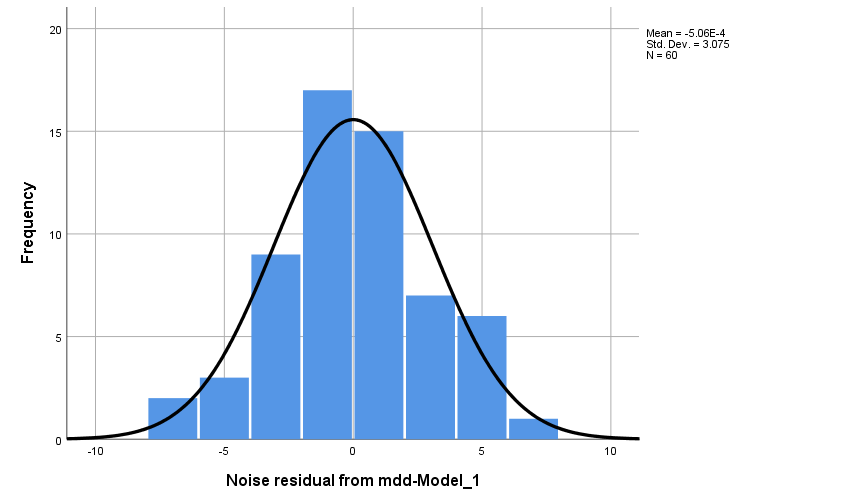

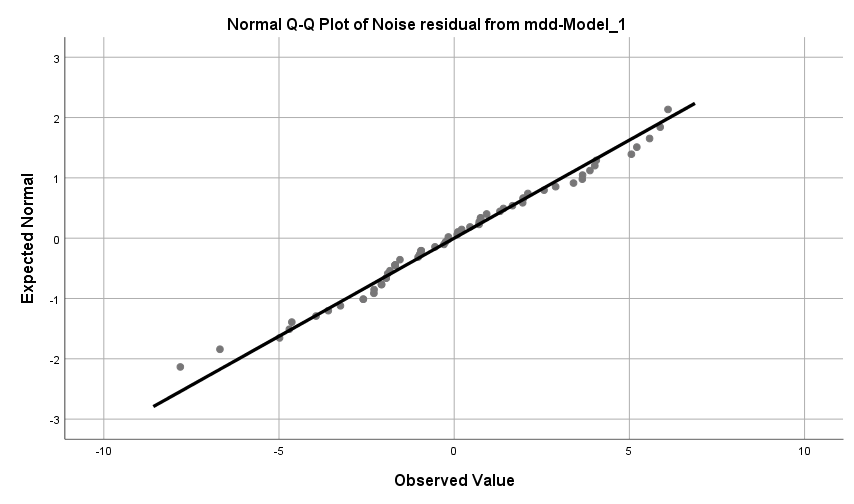


| Model | Number of Predictors | Model Fit statistics | Ljung-Box Q(18) | | | Number of Outliers |
| --- | --- | --- | --- | --- | --- | --- |
|  |  | R-squared | Statistics | DF | Sig. |  |
| MDD | 2 | .265 | 21.712 | 17 | .196 | 0 |

g) Low Mood (LD)


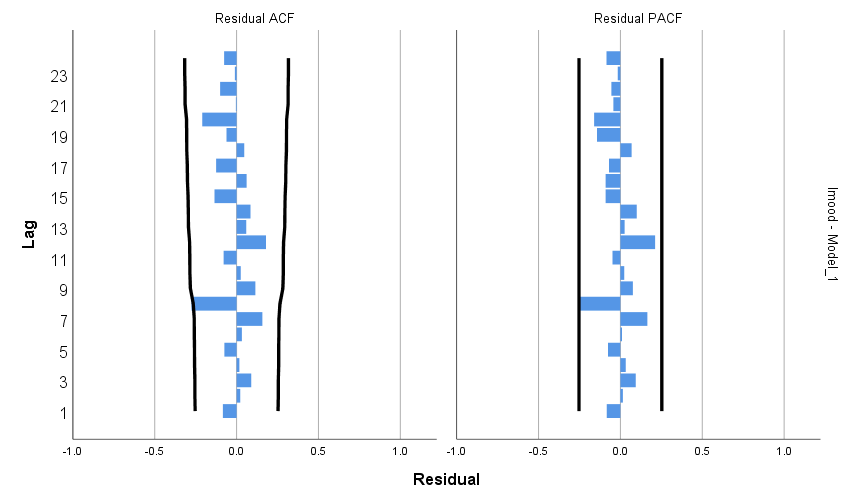

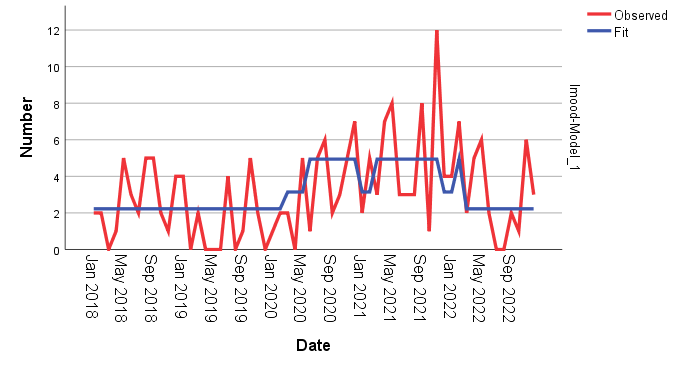


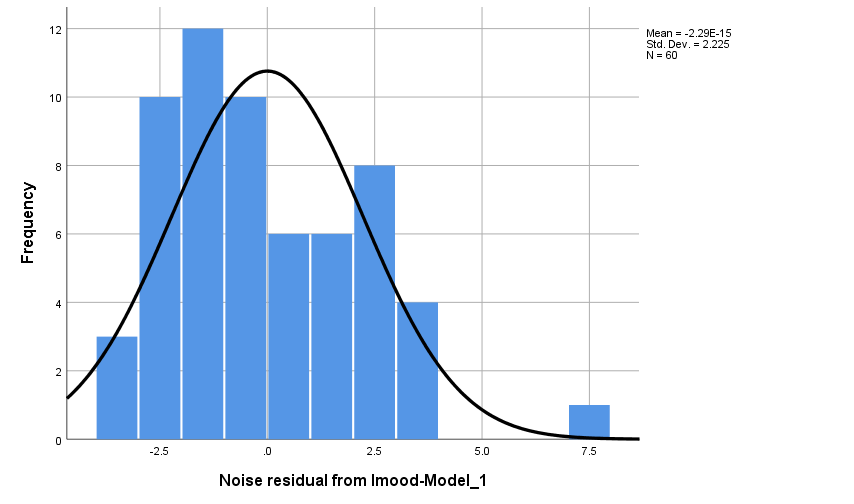

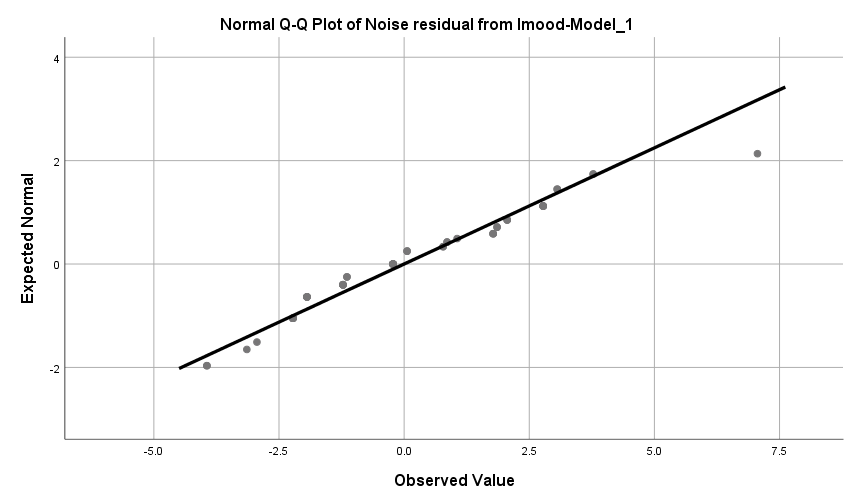


h) Oppositional Disorder (OD)


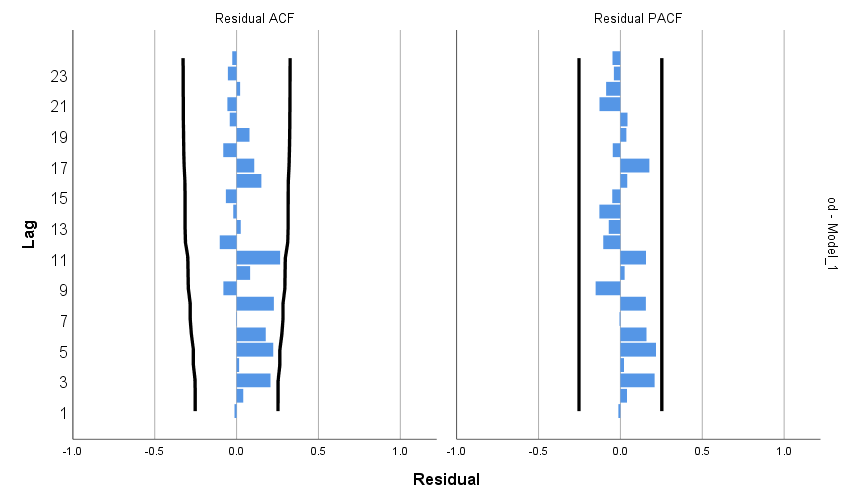

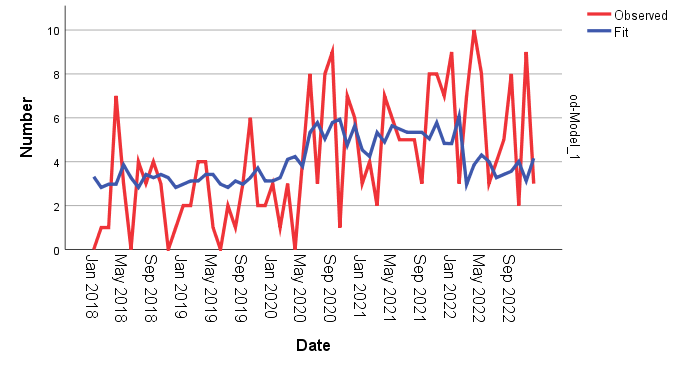


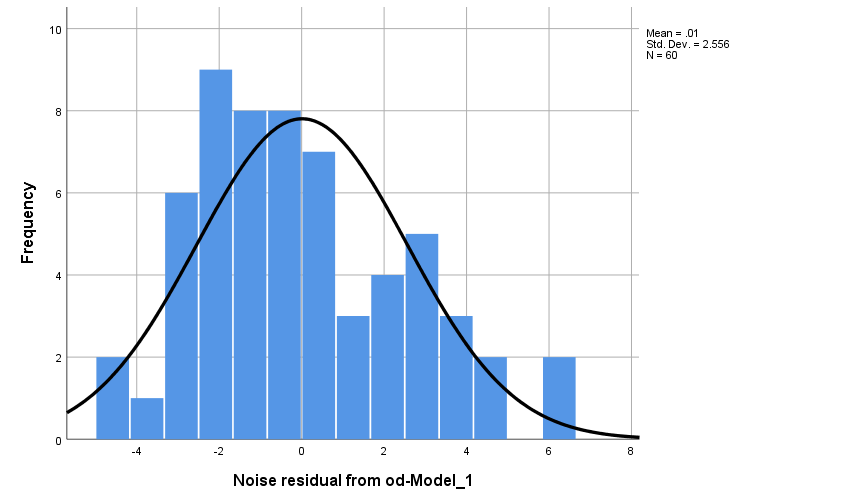

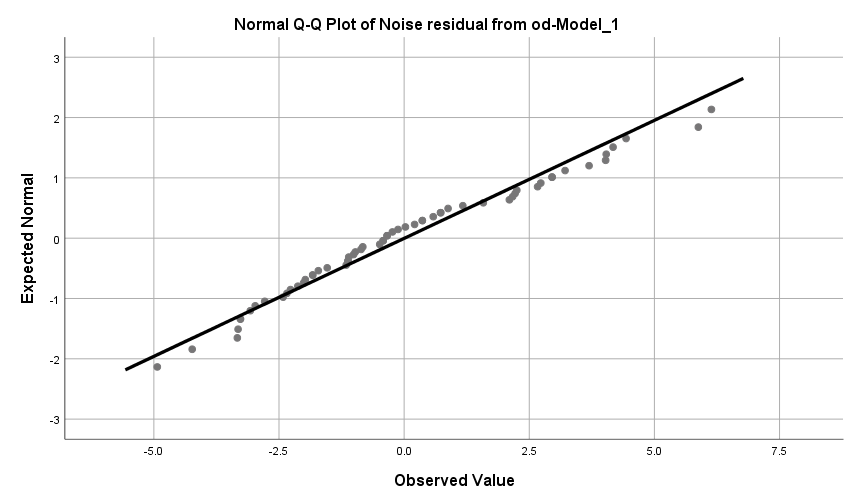


| Model | Number of Predictors | Model Fit statistics | Ljung-Box Q(18) | | | Number of Outliers |
| --- | --- | --- | --- | --- | --- | --- |
|  |  | R-squared | Statistics | DF | Sig. |  |
| OD | 2 | .145 | 23.388 | 17 | .137 | 0 |

i) ADHD


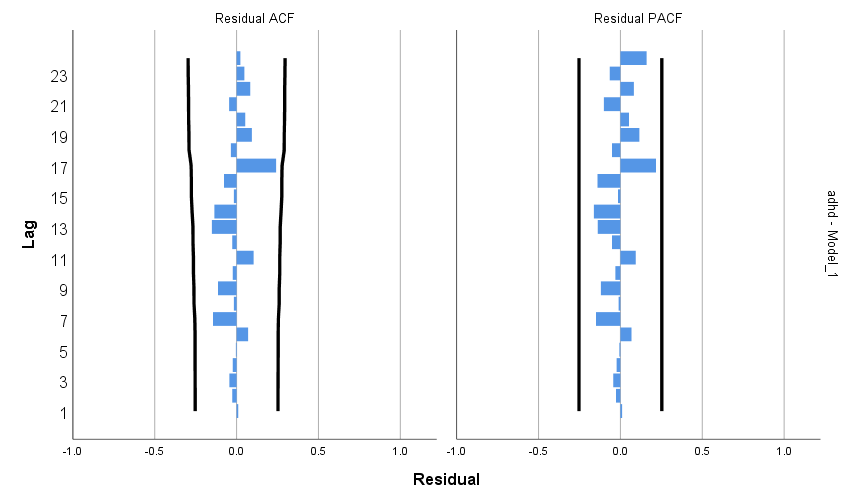

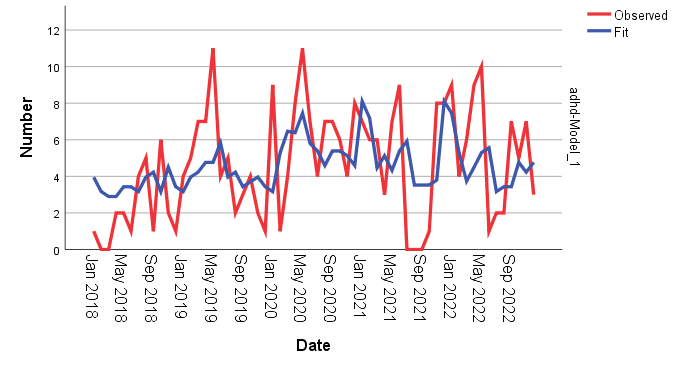


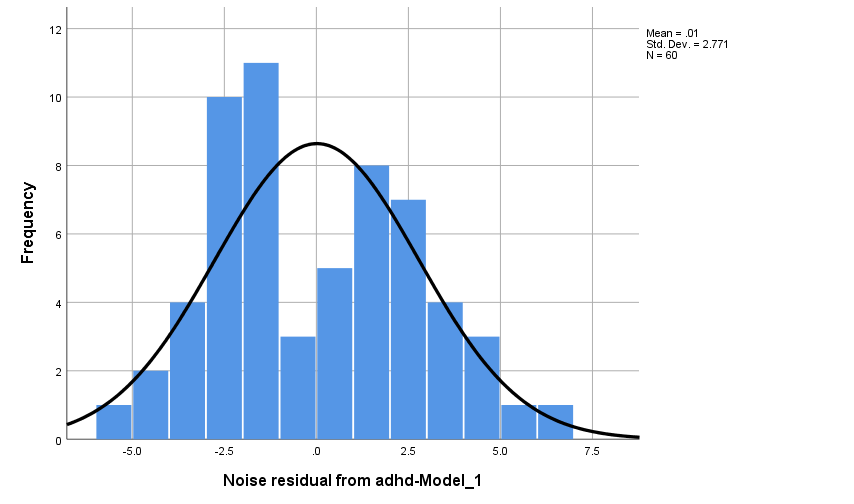

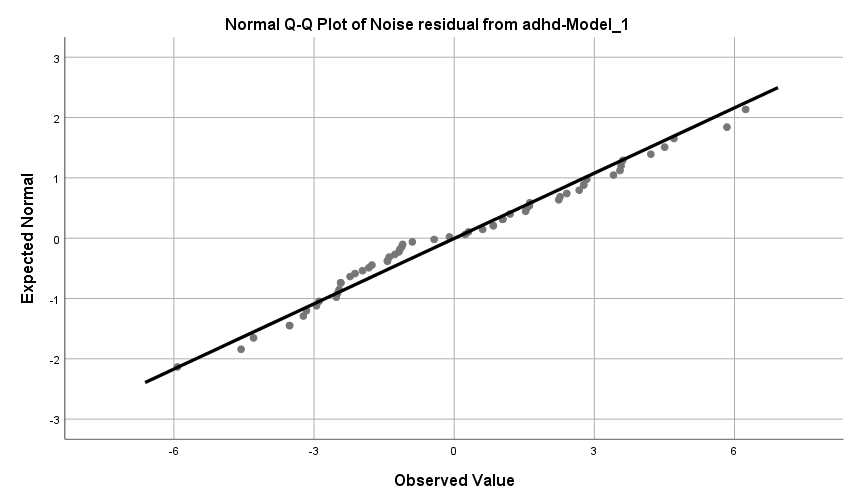


| Model | Number of Predictors | Model Fit statistics | Ljung-Box Q(18) | | | Number of Outliers |
| --- | --- | --- | --- | --- | --- | --- |
|  |  | R-squared | Statistics | DF | Sig. |  |
| ADHD- | 2 | .198 | 12.877 | 17 | .744 | 0 |

j) Autism Spectrum Disorder (ASD)


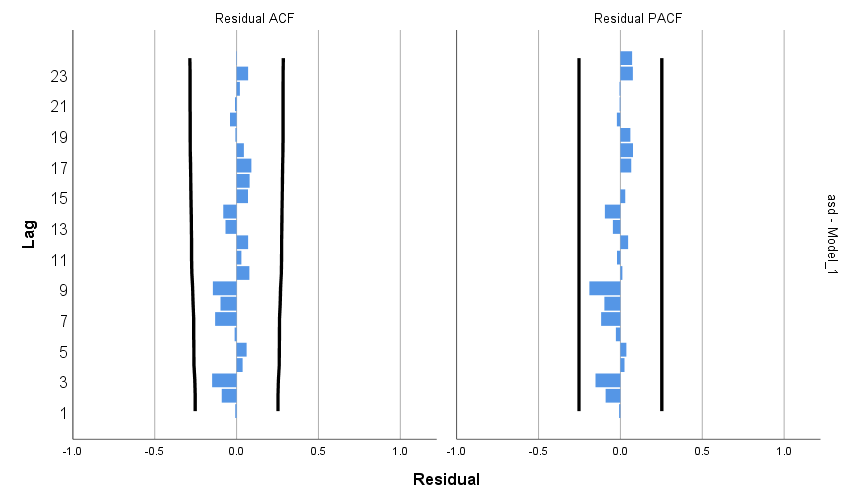

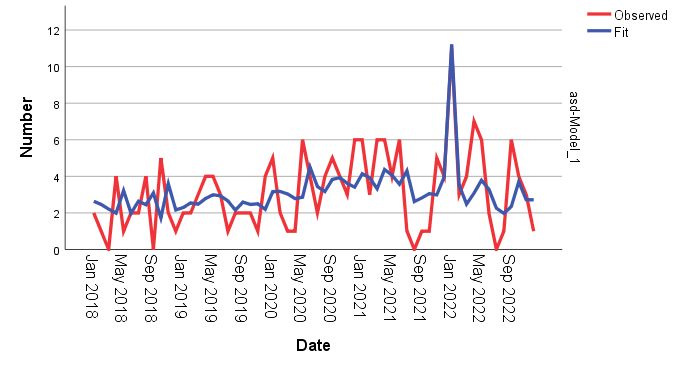


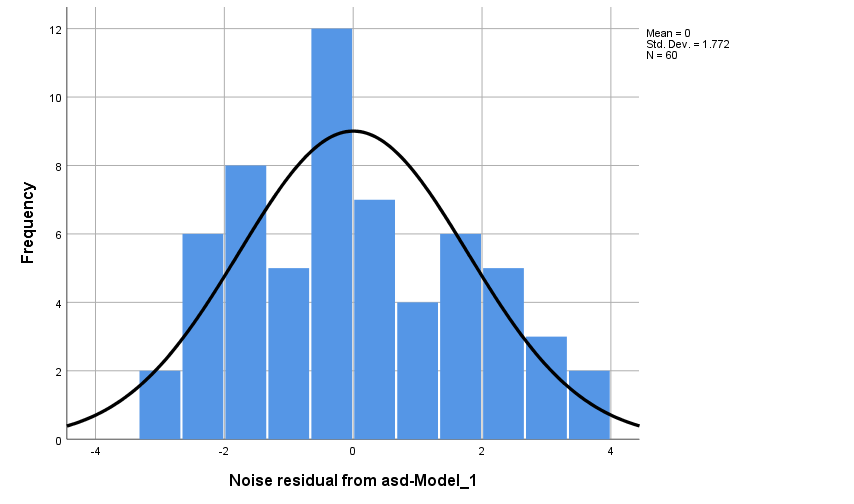

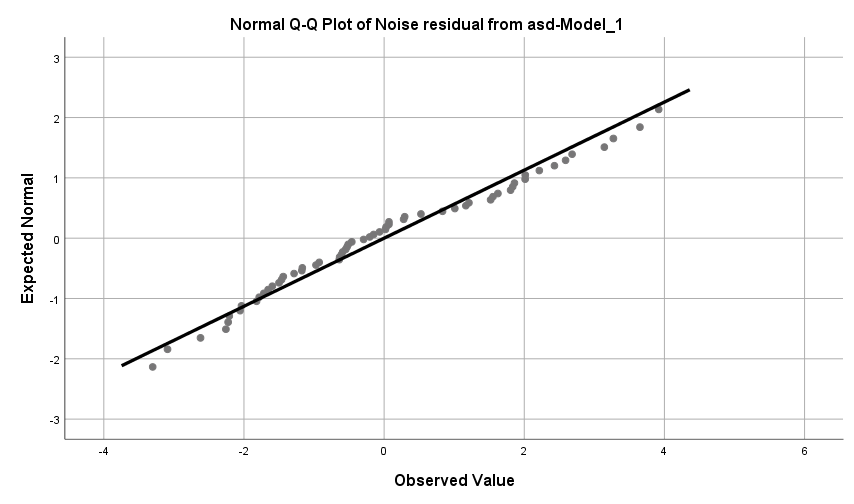


| Model | Number of Predictors | Model Fit statistics | Ljung-Box Q(18) | | | Number of Outliers |
| --- | --- | --- | --- | --- | --- | --- |
|  |  | R-squared | Statistics | DF | Sig. |  |
| ASD | 2 | .326 | 9.389 | 17 | .927 | 1 |

k) Suicidal Ideation (SI)


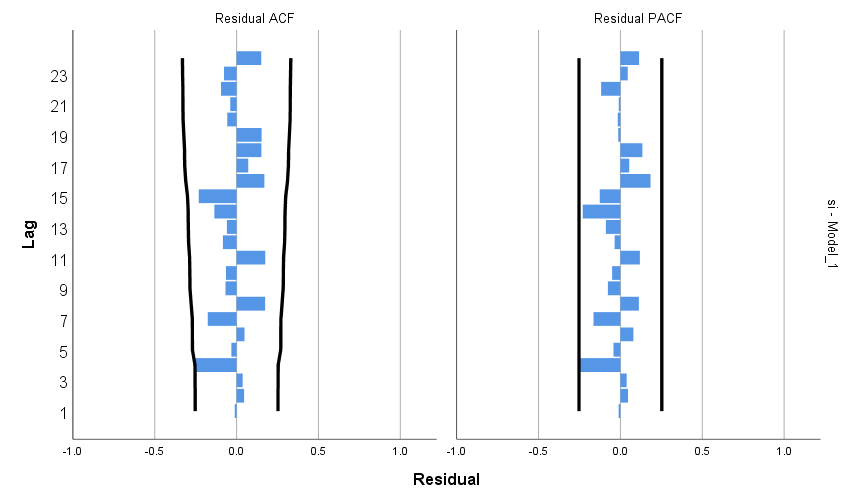

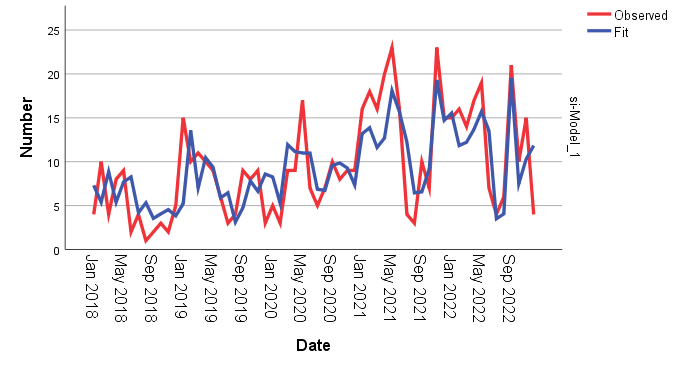


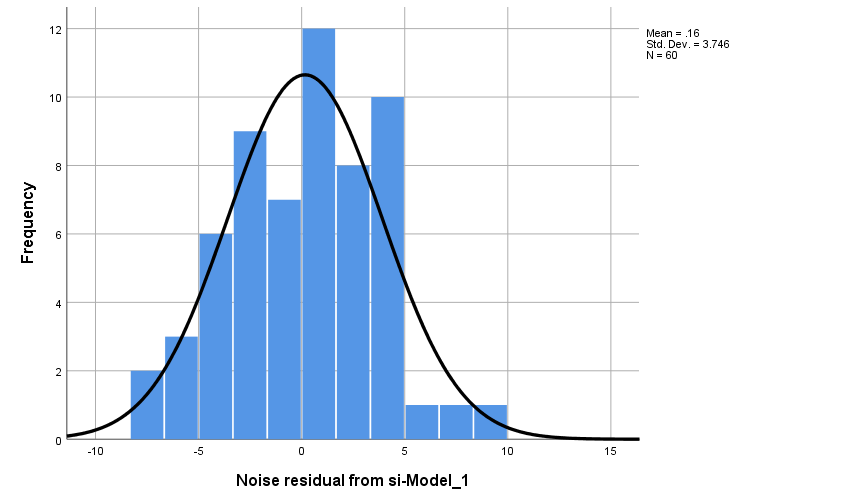

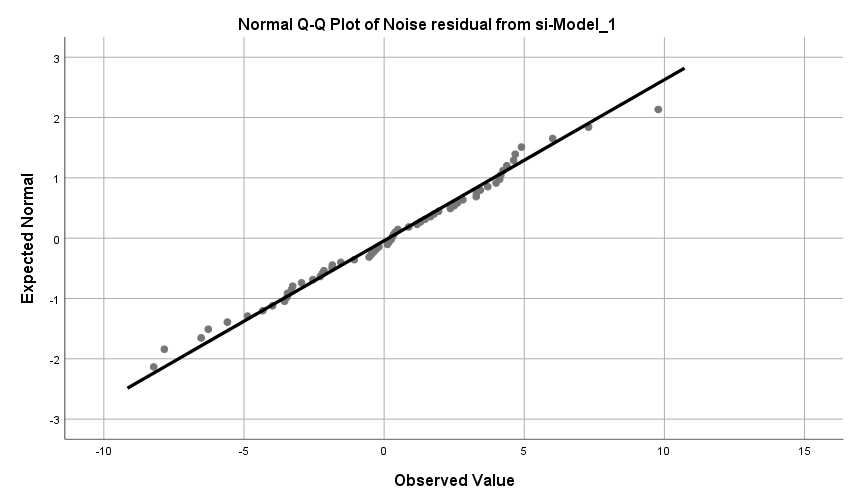


| Model | Number of Predictors | Model Fit statistics | Ljung-Box Q(18) | | | Number of Outliers |
| --- | --- | --- | --- | --- | --- | --- |
|  |  | R-squared | Statistics | DF | Sig. |  |
| SI | 2 | .581 | 23.746 | 16 | .095 | 2 |

l) Self-Harm (SH)


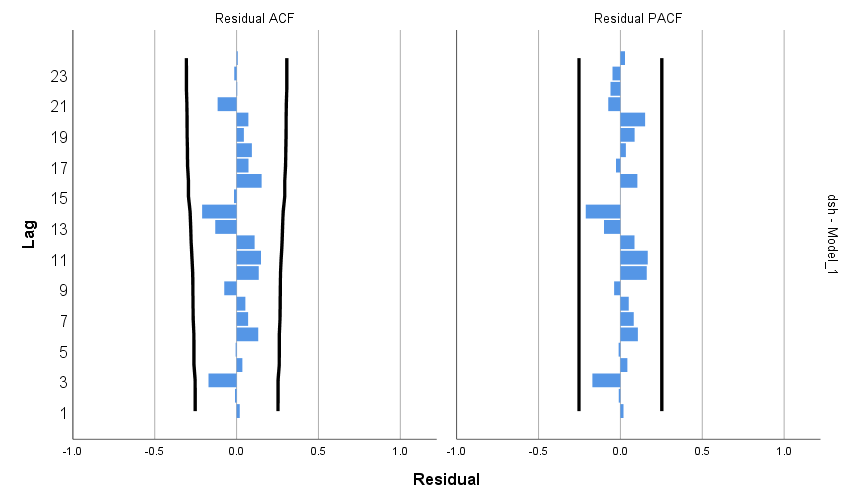

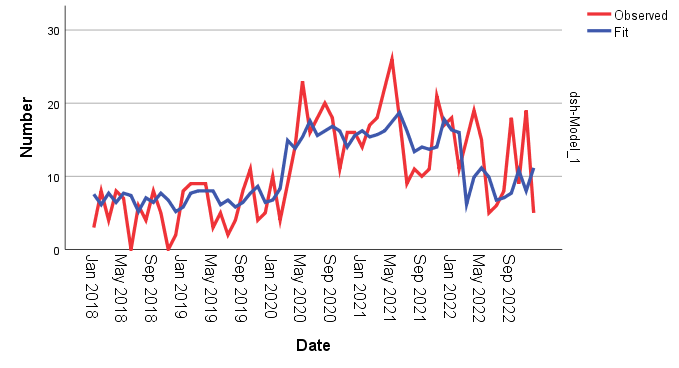


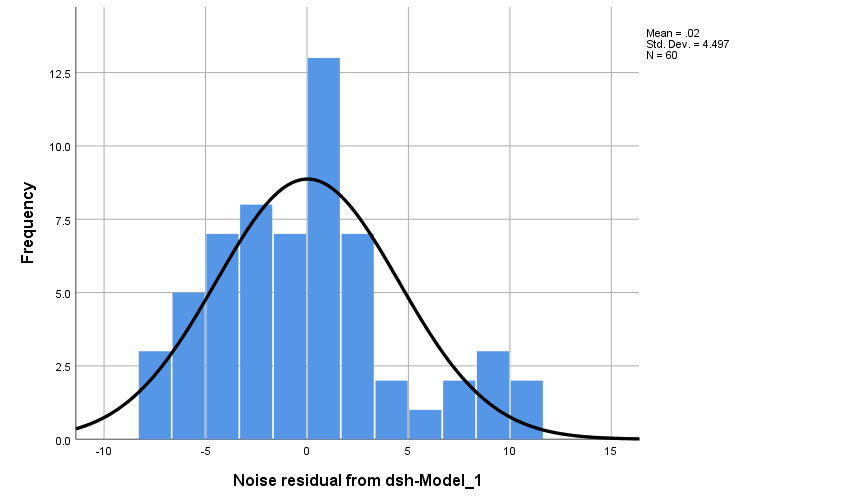

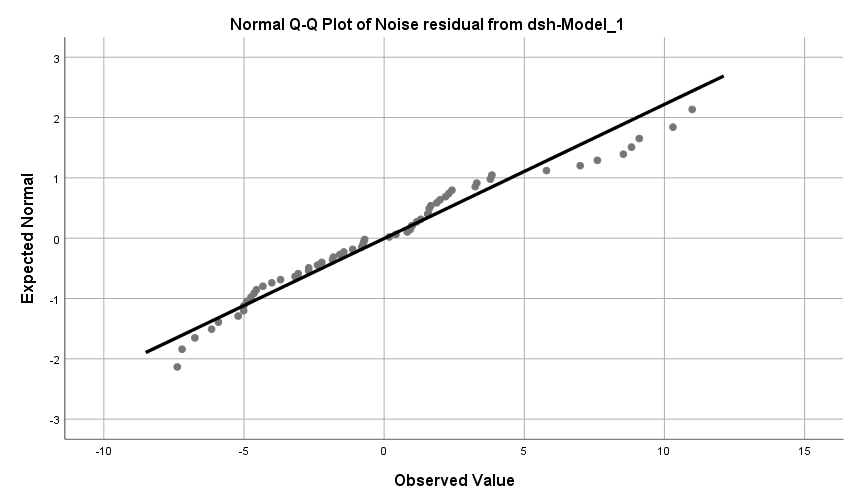


| Model | Number of Predictors | Model Fit statistics | Ljung-Box Q(18) | | | Number of Outliers |
| --- | --- | --- | --- | --- | --- | --- |
|  |  | R-squared | Statistics | DF | Sig. |  |
| SH | 2 | .501 | 16.331 | 17 | .500 | 0 |
